# Antibiofilm Coatings Based on PLGA and Nanostructured Cefepime-Functionalized Magnetite

**DOI:** 10.3390/nano8090633

**Published:** 2018-08-21

**Authors:** Denisa Ficai, Valentina Grumezescu, Oana Mariana Fufă, Roxana Cristina Popescu, Alina Maria Holban, Anton Ficai, Alexandru Mihai Grumezescu, Laurentiu Mogoanta, George Dan Mogosanu, Ecaterina Andronescu

**Affiliations:** 1 Inorganic Chemistry Department, University Politehnica of Bucharest, Bucharest 011061, Romania; denisaficai@yahoo.ro; 2 Department of Science and Engineering of Oxide Materials and Nanomaterials, Faculty of Applied Chemistry and Materials Science, University Politehnica of Bucharest, Bucharest 011061, Romania; valentina_grumezescu@yahoo.com (V.G.); oana.fufa@gmail.com (O.M.F.); roxpopescu@yahoo.co.uk (R.C.P.); alina_m_h@yahoo.com (A.M.H.); anton.ficai@upb.ro (A.F.); grumezescu@yahoo.com (A.M.G.); 3 Lasers Department, National Institute for Lasers, Plasma and Radiation Physics, Magurele RO-77125, Romania; 4 Department of Life and Environmental Physics, “Horia Hulubei” National Institute of Physics and Nuclear Engineering, Magurele RO-77125, Romania; 5 Microbiology & Immunology Department, Faculty of Biology, University of Bucharest, Bucharest 77206, Romania; 6 Research Center for Microscopic Morphology and Immunology, University of Medicine and Pharmacy of Craiova, Craiova 200349, Romania; laurentiu_mogoanta@yahoo.com; 7 Department of Pharmacognosy and Phytotherapy, Faculty of Pharmacy, University of Medicine and Pharmacy of Craiova, Craiova 200349, Romania; mogosanu2006@yahoo.com

**Keywords:** magnetite, PLGA, MAPLE, composite coatings, anti-biofilm efficiency

## Abstract

The aim of our study was to obtain and evaluate the properties of polymeric coatings based on poly(lactic-*co*-glycolic) acid (PLGA) embedded with magnetite nanoparticles functionalized with commercial antimicrobial drugs. In this respect, we firstly synthesized the iron oxide particles functionalized (@) with the antibiotic Cefepime (Fe_3_O_4_@CEF). In terms of composition and microstructure, the as-obtained powdery sample was investigated by means of grazing incidence X-ray diffraction (GIXRD), thermogravimetric analysis (TGA), scanning and transmission electron microscopy (SEM and TEM, respectively). Crystalline and nanosized particles (~5 nm mean particle size) with spherical morphology, consisting in magnetite core and coated with a uniform and reduced amount of antibiotic shell, were thus obtained. In vivo biodistribution studies revealed the obtained nanoparticles have a very low affinity for innate immune-related vital organs. Composite uniform and thin coatings based on poly(lactide-*co*-glycolide) (PLGA) and antibiotic-functionalized magnetite nanoparticles (PLGA/Fe_3_O_4_@CEF) were subsequently obtained by using the matrix assisted pulsed laser evaporation (MAPLE) technique. Relevant compositional and structural features regarding the composite coatings were obtained by performing infrared microscopy (IRM) and SEM investigations. The efficiency of the biocompatible composite coatings against biofilm development was assessed for both Gram-negative and Gram-positive pathogens. The PLGA/Fe_3_O_4_@CEF materials proved significant and sustained anti-biofilm activity against staphylococcal and *Escherichia coli* colonisation.

## 1. Introduction

Given the alarming healthcare conditions related to microbial infection, tremendous interest has been straightened towards the development of novel antimicrobials. Drug resistance phenomena related to pathogenic microorganisms [1,2,3] and the lack of specificity and side effects related to conventional pharmaceuticals [4,5,6] utilized in high amounts to treat severe infection represent one of the top concerns in scientific world. The experimental development and implementation of novel formulations with specific and selective antimicrobial activity is gathering interest worldwide from both healthcare practitioners and scientists.

The emerging technologies enable the correlation between general use non-implantable or implantable devices and complex infectious pathologies [7,8,9]. During the last decades, an impressive endeavour has been oriented towards the minimization or even elimination of microbial contamination and colonisation of medical devices. Besides the traditional sterilization strategies [10,11,12], an unconventional and attractive strategy is to develop multifunctional bioactive coatings able to limit microbial colonization of the medical devices. Recent studies showed the improved antimicrobial efficiency of thin coatings based on ceramic, polymer, composite, and hybrid materials embedded with commercial or naturally-originated antimicrobials. When designing such particular coatings for implantable devices, micro- or nanosized materials represent suitable candidates, since many of the available choices are biocompatible and can by specifically tailored for a particular therapeutic biosubstance and application.

Among the novel bioengineered polymer-based materials, poly(lactic-*co*-glycolic) acid (PLGA) represents a promising selection for unconventional biotechnology and personalized biomedicine applications. Great attention has been paid towards the multi-functionality of poly(lactide-*co*-glycolide)-based materials, thanks to the indisputable properties of this copolymer, including the physicochemical versatility (which enables a wide variety to design and develop novel materials, multitudinous mixtures with organic or inorganic structures, adjustable compositional and microstructural features and tunable degradation, facile and beneficial interactions with simple biosubstances or complex biomacromolecules) and excellent biological behaviour (biocompatibility and biodegradability, approval from healthcare specialized agencies etc.) [13,14,15].

The suitable tailoring of PLGA-based materials contributes to the successful synthesis of novel and performant biomaterials for different biomedical-related applications, including unconventional platforms for targeted, controlled and specific drug administration [16,17,18,19,20], genuine systems for selective and specific diagnosis [21,22,23], multifunctional materials for tissue engineering [24,25,26,27] and innovative personalized therapeutic strategies [28,29,30,31,32].

Magnetite-based nanomaterials are of a great interest in recent years, mainly because of the great biocompatibility, magnetic properties and since the peculiar size-related features of iron oxide nanomaterials strongly recommend them for various biotechnological and biomedical applications. Thanks to their specific physicochemical properties and biological behaviour, magnetite (Fe_3_O_4_) nanoparticles represent a novel and challenging alternative for conventional diagnosis [33,34,35], imaging [36,37,38] and therapy [39,40,41,42].

In particular, Fe_3_O_4_-based nanostructures have been evaluated as promising materials for the development of unconventional antimicrobials. Different studies reported the potentiating effects of magnetite nanoparticles with respect to the antibacterial effects exhibited by various commercial drugs [43,44] and natural-derived substances [45] against planktonic and biofilm-organized bacteria. Also, positively charged nanosystems based on magnetite core and chitosan shell loaded with antibiotics, such as Kanamycin or Neomycin resulted in a significant potentiated bactericidal effect, when compared to pristine antimicrobial drugs [46]. Thin films based on Kanamycin-functionalized Fe_3_O_4_ nanosystems were evaluated as biocompatible materials with significant inhibition effects against the development of both Gram-negative and Gram-positive bacterial biofilms [47]. Also, biocompatible coatings based on poly(3-hydroxybutyrate-*co*-3-hydroxyvalerate)-polyvinyl alcohol (PHBV-PVA) microspheres embedded with eugenol-functionalized Fe_3_O_4_ nanoparticles proved to significantly affect the bacterial contamination and biofilm development [48].

The aim of our study was to synthesize biocompatible and antibacterial coatings based on poly(lactic-*co*-glycolic) acid and magnetite nanoparticles functionalized with a fourth-generation antibiotic (Cefepime) in order to obtain improved surfaces, useful for various biomedical applications.

## 2. Materials and Methods

### 2.1. Materials

Anhydrous ferric chloride (FeCl_3_, >99.99% trace metal basis), ferrous sulphate heptahydrate (FeSO_4_·7H_2_O, >98%) and ammonium hydroxide solution (25% NH_3_ in H_2_O) were purchased from Sigma-Aldrich Chemie GmbH (Darmstadt, Germany). The same supplier was selected to procure the Cefepime hydrochloride (>98% purity) and the poly(lactic-*co*-glycolic acid) copolymer (PLGA) with 50:50 lactide to glycolide molar ratio. Analytical graded acetone (C_6_H_6_O), ethanol (C_2_H_6_O), and dimethyl sulfoxide (DMSO, C_2_H_6_OS) were also purchased from Sigma-Aldrich Chemie GmbH (Darmstadt, Germany).

The after-mentioned supplier was also selected to procure the substances required for the biological assays, respectively Dulbecco’s Modified Eagle Medium (DMEM), pristine and agar-containing Luria Broth (LB) medium, foetal bovine serum (FBS), physiological sterile phosphate buffered saline (PBS), penicillin/streptomycin/neomycin (P/S/N) antibiotic mixture. Vybrant^®^ MTT Cell Proliferation Assay Kit was purchased from Invitrogen (Thermo Fischer Scientific, Waltham, MA, USA).

Human-derived amniotic fluid stem cells (AFSCs), as well as *Escherichia coli* ATCC 15224 and *Staphylococcus aureus* ATCC 25923 bacterial strains were obtained from the American Type Culture Collection (ATCC, Manassas, VA, USA).

The required glass and high purity (100) silicon substrates (1 cm^2^) were purchased from a local supplier.

### 2.2. Synthesis Methods

#### 2.2.1. Synthesis of Fe_3_O_4_@CEF Nanoparticles

The obtaining of iron oxide nanoparticles functionalized with Cefepime was experimentally performed by using the versatile co-precipitation synthesis strategy. In this respect, two aqueous solutions containing the metallic precursors and the therapeutic agent were needed, respectively. The first solution resulted by dissolving both the FeCl_3_ and FeSO_4_·7H_2_O in 200 mL of ultrapure water, while the second solution resulted by suspending the Cefepime hydrochloride in 400 mL of ultrapure water together with 8 mL of NH_3_ solution. The metallic precursor solution was further added drop-by-drop into the drug-containing solution under continuous magnetic stirring, at room temperature. Once the pipetting process ended, the resulted solution was filtered and the collected precipitate product was subjected to a triple washing treatment with ultrapure water. The as-obtained filtering product was afterwards dried at 40 °C for 6 h, using a conventional drying stove. A reduced amount of the as-obtained powdery sample was further used for physicochemical and biological investigation, while the most part of the Fe_3_O_4_@CEF powder was subjected to the laser processing, in order to synthesise the composite multifunctional coatings. The same co-precipitation method was used to experimentally obtain pristine Fe_3_O_4_-based powder.

#### 2.2.2. Synthesis of PLGA/Fe_3_O_4_@CEF Coatings

The previously synthesized Cefepime-functionalized Fe_3_O_4_ powder was mixed with PLGA and the resulted PLGA/Fe_3_O_4_@CEF mixture was further dispersed in 99% DMSO and 1% dichloromethane solution to obtain a colloidal suspension of 0.7% (*w*/*v*) concentration. Subsequently, the as-obtained suspension was frozen in liquid nitrogen during 30 min. The resulted frozen composite materials were placed onto the cooling system of the reaction chamber within the Matrix Assisted Pulsed Laser Evaporation (MAPLE) experimental setup and irradiated with a laser beam generated by a KrF* excimer laser source (λ = 248 nm, τ_FWHM_ = 25 ns) from Lambda Physics, Coherent. Prior to the MAPLE experiments, all glass and silicon substrates were subjected to a successive cleaning treatment with acetone, ethanol and deionized water under ultrasonic conditions. The deposition experiments were performed at room temperature conditions with 0.1 Pa background pressure inside the reaction chamber, by keeping constant the target-substrate distance (4 cm), the frequency of the laser beam (10 Hz) and the applied number of pulses (30,000). In order to estimate the optimal parameters of the laser processing, the experiments were performed by using various fluences of the laser beam (300, 400 and 500 mJ/cm^2^). Moreover, the same colloidal suspension was used for the dropcast experiments, which were performed on Fourier transform infrared spectroscopy (FT-IR) transparent silicon substrates.

### 2.3. Physicochemical Characterisation

#### 2.3.1. Grazing Incidence X-ray Diffraction (GIXRD) 

The GIXRD analysis was performed by using an Empyrean diffractometer equipped with hybrid monochromator and PIXcel^3D^ detector, from PANalytical (Almelo, The Netherlands). In this respect, a small amount of the synthesized powdery material was investigated by using the CuKα radiation (λ = 1.5418 Å) of the instrument, which enabled individuals scans at every 3 seconds between 5° and 80° 2θ scattering angles, at 0.5° incidence angle.

#### 2.3.2. Thermogravimetric Analysis (TGA) 

Comparative thermal analysis was performed by using a DTG-TA-50H equipment from Shimadzu (Torrance, CA, USA). Small amounts of the resulted powdery samples were placed in alumina crucibles and the concerned thermal tests were performed in dried synthetic air (80% N_2_ and 20% O_2_) from room temperature up to 900 °C, by using a 1 °C/min heating rate.

#### 2.3.3. Transmission Electron Microscopy (TEM)

The TEM data were collected by using the Tecnai^TM^ G2 F30 S-TWIN high resolution transmission electron microscope from FEI Company (HILLSBORO, OR, USA), equipped with selected area electron diffraction (SAED) instrument. The specific point and line resolutions of to the microscope are 2 and 1 Å, respectively. Before TEM investigation, a small amount of the Fe_3_O_4_@CEF sample was dispersed in ethanol, sonicated for 15 min, placed onto the carbon-coated cooper grid and dried at room temperature.

#### 2.3.4. Infrared Microscopy (IRM)

Comparative infrared studies of dropcast and laser processed samples were performed by using the reflection mode of the Nicolet^TM^ iN10 MX FT-IR (Thermo Fischer Scientific, Waltham, MA, USA) microscope with MCT liquid nitrogen cooled detector, purchased from (Thermo Fischer Scientific, Waltham, MA, USA). 32 individual scans were recorded for each sample between 4000 and 700 cm^−1^ wavenumbers at 4 cm^−1^ resolution, co-added and converted to absorbance spectra by using the OMNICPicta^TM^ software package (Version 1, Thermo Fischer Scientific, Waltham, MA, USA).

### 2.4. Biological Evaluation

#### 2.4.1. Biocompatibility Evaluation

In order to experimentally estimate the biocompatibility of the synthetized composite coatings, we performed in vitro quantitative assays on amniotic fluid-derived stem cells (AFSCs). The selected viability assay involves the optical correlation between the number of viable cells and the amount of formazan (an insoluble purple product resulted after the mitochondria-mediated reduction of yellow MTT tetrazole (3-(4,5-dimethylthiazol-2-yl)-2,5-diphenyltetrazolium bromide) in active cells). Prior to the concerned assays, all the samples were sterilized by UV light exposure. 

For the quantitative biocompatibility evaluation of the synthesized composite coatings, reduced amounts of the PLGA/Fe_3_O_4_@CEF samples were obtained by scratching a 0.25 cm^2^ area of the MAPLE deposited glass substrates and placed in 96-well plates. Subsequently, 3000 AFSCs were inoculated in each well in the presence of 300 μL DMEM medium supplemented with 10% FBS and 1% P/S/N mixture and cultivated under standard conditions (37 ± 2 °C, 5 ± 1% CO_2_, more than 90% humidity) for 72 h. Within our experiments, the controls were represented by stem cells cultivated in the absence of the Fe_3_O_4_-based coatings, in the same conditions. According to the manufacturer’s guidelines, the viability assay was performed at various time intervals in triplicate experiments. After the incubation period, 10 μL of 12 mM MTT solution were added in each well and the as-treated specimens were incubated in dark conditions for 4 h. Supplementary 4 h incubation period was required after we added in each well 100 μL from the second component within the assay kit (SDS-HCl solution). The absorbance values corresponding to the as-resulted solutions were read by spectrophotometric means at 570 nm, using in this respect a Mithras LB 940 instrument from Berthold Technology (Bad Wildbad, Germany).

#### 2.4.2. Antimicrobial Evaluation

##### Antimicrobial Evaluation of the Core-Shell Nanoparticles 

For establishing the antimicrobial effect against planktonic cultures in a dose-dependent manner and to estimate the drug-release ability of the nanosystem we have utilized and adapted microdilution protocol, described by Grumezescu and co-workers [46]. The sterile broth was added in sterile 96 well plates and binary dilutions of each tested compound/nanosystem were performed in a final volume of 150 μL. After realizing the binary dilutions, 15 μL of microbial suspension adjusted to an optical density of 0.5 McFarland (1.5 × 10^8^ CFU/mL) were added in each well. The growth inhibition was established by spectrophotometric measurement (Abs 600 nm) of the optical density (OD) of the obtained cultures.

##### Anti-Biofilm Effect of the Coatings

The antimicrobial and anti-biofilm properties of the synthesized composite coatings were in vitro assessed by the quantitative estimation of colony forming units (CFU/mL), which are directly related to the development of bacterial biofilm on the obtained surfaces. After the UV sterilisation treatment of the samples (20 min exposure on each side), the assays were performed by using a Gram negative (*Escherichia coli*) and a Gram positive (*Staphylococcus aureus*) bacterial strains. In this respect, the concerned specimens (PLGA/Fe_3_O_4_@CEF-coated and uncoated glass slides) were placed in 6-well plates in the presence of 2 mL of LB medium and inoculated with 50 μL bacterial suspensions of 0.5 McFarland standard density (1.5 × 10^8^ CFU/mL). After standard incubation of the plates for 24 h, the samples were washed with PBS (phosphate buffered saline solution) and the culture medium was removed and replaced with fresh LB medium. After the required incubation periods (24, 48 and 72 h), all the specimens were washed with PBS and placed in individual sterile tubes in the presence of 1 mL PBS. In order to disperse the biofilm-forming bacterial cells, each tube was vigorously vortexed for 30 seconds. Serial ten-fold dilutions were prepared from each resulted solution and 10 µL from the obtained dilutions were transferred to Petri plates containing LB agar, for viable cells count assay [49,50]. All the experiments were performed in triplicate. 

#### 2.4.3. In Vivo Biocompatibility and Biodistribution

Regarding the histological aspects of the main organs of mouse animal model, the in vivo distribution of Fe_3_O_4_@CEF was investigated. The experimental protocol was applied in accordance with the European Council Directive No. 86/609 (22 November 1986), the European Convention for the protection of vertebrate animals used for experimental and other scientific purposes (2 December 2005) and Law No. 43 (11 April 2014) on the protection of animals used for scientific purposes, adopted by the Parliament of Romania. The study was approved by the Bioethics Commission of the University of Medicine and Pharmacy of Craiova (Report No. 118/27.05.2015). BALB/c mice of three months age were aseptically injected with 100 μL of nanostructure dispersion (1 mg/mL) prepared in physiological saline and pre-sterilized by UV irradiation for 30 min. Intravenous administration was performed in the jugular left vein using a catheter under general anaesthesia with ketamine/xylazine intraperitoneally injected. Control mice were injected under the same conditions with 100 μL of physiological saline. Throughout the experiment, the animals were kept under standard temperature, humidity, lighting, food and water (ad libitum). At 2 days and 10 days after the inoculation, the animals were euthanized after general anaesthesia for the purpose of harvesting internal organs (brain, liver, myocardium, pancreas, lung, kidney, spleen). Immediately after harvesting, organ fragments were washed in TFS (saline phosphate buffer) for blood removal, preserved in 10% formaldehyde solution at room temperature for 72 h and subsequently processed by inclusion in paraffin for the purpose of carrying out studies of microscopic morphology. For histological analysis of the nanostructures, in paraffin blocks with biological samples were obtained serial sections with a thickness of 4 μm, using a MICROM HM355s rotary microtome (MICROM International GmbH, Walldorf, Germany) equipped with a transfer system (STS, microM) (Thermo Fischer Scientific, Waltham, MA, USA). The cross-sections were placed on histological blades treated with poly-L-lysine (Sigma-Aldrich, Munich, Germany). After applying the Haematoxylin-Eosin (HE) classical staining technique, the sections were evaluated and photographed on a Nikon Eclipse 55i optical microscope equipped with Nikon DS-Fi1 CCD (Nikon Instruments, Apidrag, Romania). The acquisition, storage and image processing was done using the Image ProPlus 7 AMS software package (Media Cybernetics Inc., Marlow, Buckinghamshire, UK).

## 3. Results

### 3.1. Physicochemical Characterisation of Fe_3_O_4_@CEF Nanoparticles

**Figure 1:**
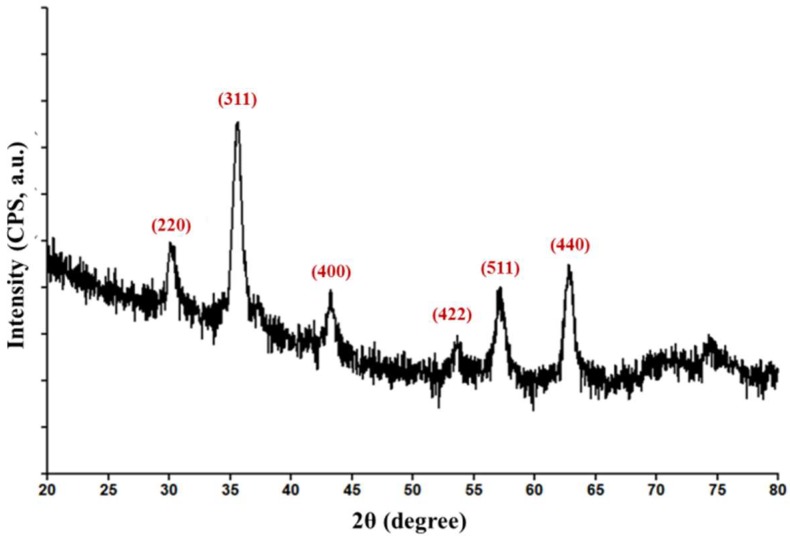
Diffractogram of Fe_3_O_4_@CEF powdery sample.

Relevant data regarding the purity and the crystallinity of the synthesized powdery sample was obtained by considering the GIXRD results, depicted in Figure 1. 

The obtained pattern reveals the presence of broad diffraction maxima at 2θ angles of 30°, 35.80°, 44.10°, 54°, 57.50°, and 63.80°. This observation indicates the successful synthesis of ultra-fine particles with particular reduced crystallite size. According to the available standard charts and to the literature data [47,51], the previously identified diffraction interferences correspond to the (200), (311), (400), (422), (511), and (440) diffraction planes of face centred cubic crystallographic system of magnetite. When compared to the standard International Centre of Diffraction Data (ICDD) card No.19-0629, no additional peaks corresponding to other crystalline phases and no angular position changes of the identified diffraction maxima are present. Thus, the obtained diffractogram indicates the successful synthesis of high purity magnetite-based powdery sample.

Significant qualitative and quantitative information regarding the thermal behaviour of the Fe_3_O_4_-based powders and the amount of therapeutic agent embedded within the functionalized samples was obtained by gathering the mass loss and the nature of the concerned process from TG (thermogravimetric analysis) and DSC (differential scanning calorimetry) curves, respectively.

According to the obtained derivatograms (not shown), corresponding to both pristine (and functionalized Fe_3_O_4_-based samples, the presence of weak endothermic peaks occurred below 150 °C was noticed. The as-identified peaks are accompanied by mass changes of 0.81% and 2.39%, respectively, and can be assigned to the evaporation of humidity water from the powdery samples [52,53]. Heating the pristine sample up to 400 °C resulted in a weak endotherm process corresponding to a 1.27% mass change, which may be assigned to the thermal removal of abundant hydroxyl groups attached onto the surface of the iron oxide nanoparticles. In this particular case, the endothermic maximum identified at 139.2 °C is specifically related to the removal of intrinsic moisture of the sample. In comparison, the same thermal treatment applied to the functionalized sample resulted in two additional endothermic processes, accompanied by a total mass change of 2.95%. For what concerns the prominent endothermic maximum identified at 218.7 °C, one can say that it is specifically assigned to the thermal degradation of the organic backbone within the antibiotic agent used during the synthesis process. For the weak endothermic process occurred between 280 and 400 °C, one can presume the same thermic removal of hydroxyl groups as in the case of pristine Fe_3_O_4_-based sample. In both cases, the presence of strong endothermic processes occurred at 558.9 and 540.1 °C, respectively, can be specifically assigned to the isomorphic transition of magnetite into γ-maghemite [54,55]. The residual mass (marked in green) corresponding to the pristine magnetite and Fe_3_O_4_@CEF samples is 97.19% and 93.66%, respectively. One can thus conclude the successful synthesis of Cefepime-functionalized magnetite powder with an estimated amount of the embedded antibiotic of 3.53 ± 0.1%.

**Figure 2:**
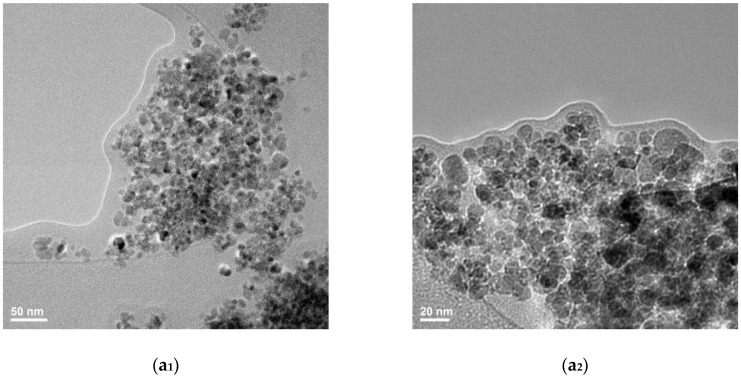

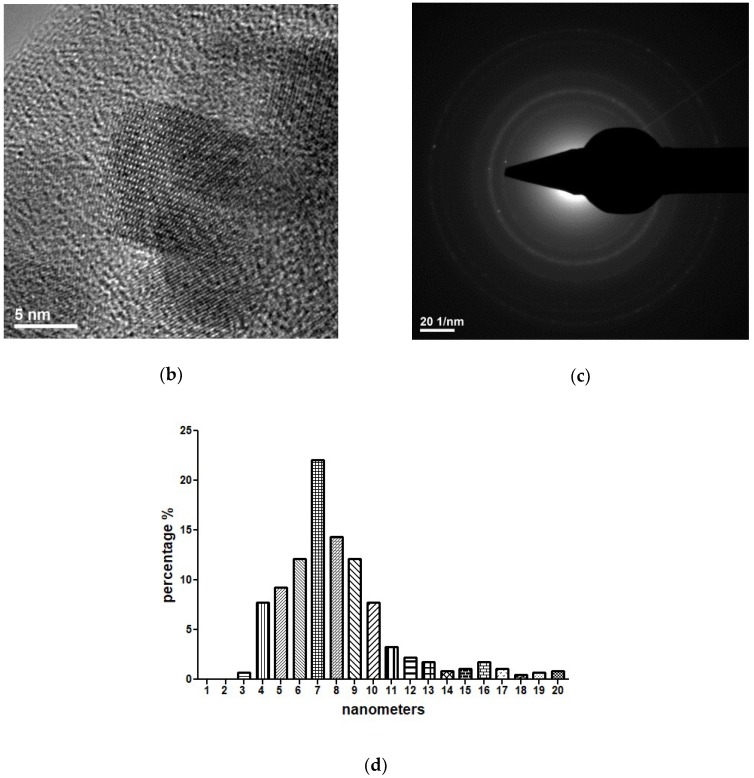
Transmission electron microscopy (TEM) = (**a_1_,a_2_**) and HR-TEM = (**b**) images and selected area electron diffraction (SAED) pattern (**c**) of Fe_3_O_4_@CEF powdery sample. Figure (**d**) reveals the percentage of nanoparticle size in the analysed samples.

From the TEM (Transmission electron microscopy) images in Figure 2 one can identify particular aspects related to the intimate microstructure of the Cefepime-functionalized Fe_3_O_4_-based samples. At higher magnification, in HR-TEM (High resolution-transmission electron microscopy) (Figure 2b) one can notice the presence of a thin bright region that surrounds the iron oxide particles. This is the result of a thin organic layer of Cefepime formed during the synthesis process onto the surface of magnetite particles and is responsible for the reduced agglomeration tendency of the Fe_3_O_4_@CEF systems (thanks to the beneficial spacer role of the antibiotic). Such observation is of critical significance, if we consider the future requirements of Fe_3_O_4_ particles regarding their stability in polar solvents. Moreover, the obtained micrographs confirm the preferential sphere-like shape of the synthesized particles, but also the nanosized dimensions, since the mean size of the magnetite particles was evaluated as ~7 nm. Furthermore, the collected SAED spectra (Figure 2d) are in good agreement with the previously discussed X-ray Diffraction data regarding the presence of face centred cubic magnetite as the sole crystalline phase within the synthesized sample.

**Figure 3:**
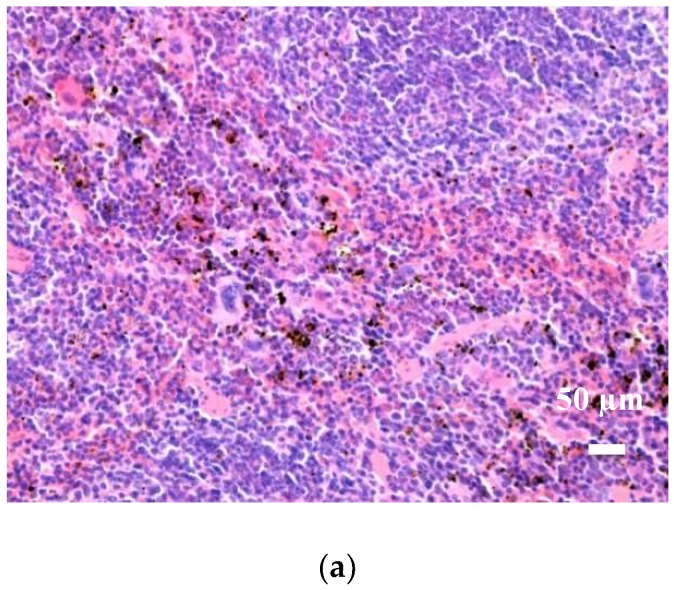

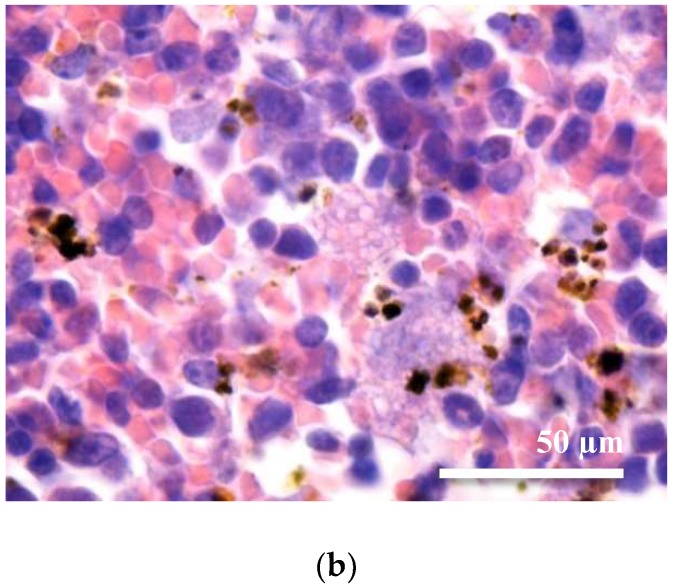
Optical micrographs of splenic tissue harvested at 10 days after injection of Fe_3_O_4_@CEF nanoparticles at 200× (**a**) and 1000× (**b**) magnification.

In vivo biodistribution results revealed that the Fe_3_O_4_@CEF nanosystems are transported through the blood flow and preferentially localized in certain organs (Figure 3).

In the brain and pancreas tissues, the Fe_3_O_4_@CEF nanoparticles were absent, regardless the evaluation period. At the hepatic level, two days later, low-level nanoparticles were seen in both blood vessels and Kuppfer cells at the periphery of liver sinusoidal capillaries. The density of nanoparticles in Kuppfer cells was variable from one cell to another, being directly proportional to the size of the sinusoidal capillary in the hepatic parenchyma. At the pulmonary level, the Fe_3_O_4_@CEF nanoparticles were mainly detected in perivascular macrophages and intracellular septa. The nanoparticle density was different depending on the cell type. The highest density was found in the perivascular macrophages and the lowest in the macrophages in the interalveolar septa.

At renal level, for the Fe_3_O_4_@CEF sample, the nanoparticles were identified in low concentration, only in the blood vessels. In the rest of the renal parenchyma (glomeruli, renal tubules, renal stroma) no nanoparticles were found. Regarding the spleen, the histological evaluation evidenced that the Fe_3_O_4_@CEF nanoparticles were highlighted only in the red pulp. In the white pulp, nanoparticles were absent. However, white pulp hypertrophy was observed, due to the fact that antibiotic-functionalized magnetite nanoparticles stimulated the formation of multilobular nucleus macrophages.

In vivo evaluation of biocompatibility and biodistribution on mice 10 days after injection revealed that in the brain, liver, myocardium, pancreas, lungs and kidneys were negative for the presence of Fe_3_O_4_@CEF nanoparticles, since neither morphological modifications nor functional alterations were identifies at these levels.

In the spleen, for Fe_3_O_4_@CEF, 10 days later, the nanoparticles were noticed only in the red pulp, in a higher concentration than that observed in the splenic samples harvested at two days. In the splenic white pulp, nanoparticles were absent. And in this case, white pulp hypertrophy was observed, due to the fact that nanoparticles stimulated the formation of multilobular nucleus macrophages.

**Figure 4:**
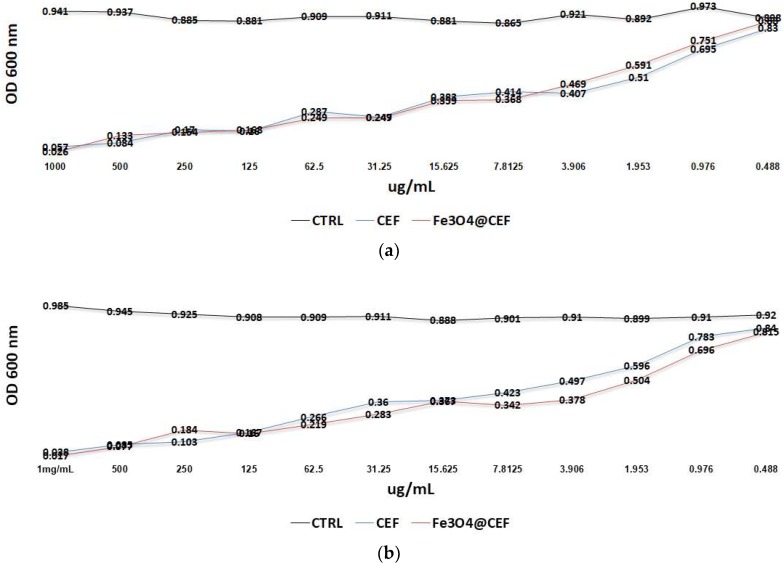
Graphic representation of *E. coli* (**a**) and *S. aureus* (**b**) growth for 24 h in the presence of different concentrations of plain Cefepime hydrochloride (CEF) and nanosystem embedded cefepime hydrochloride (Fe_3_O_4_@CEF).

In order to analyse the antimicrobial effect of different concentrations of nanoparticles and also to predict the in vitro drug release capacity we have used a microdilution model [46]. The results demonstrated that the obtained nanosystem has inhibitory effects against microbial growth in a dose dependent manner, being active at much lower concentrations, as compared with plain CEF solution, utilized at the same concentration. Positive results were obtained in the case of *S. aureus*, where, bacteria cells grow slower with an average of ~10% in the presence of Fe_3_O_4_@CEF at concentrations between 7.8 and 0.9 μg/mL. This result suggests that the obtained nanosystem potentates the activity of the active drug, most probably by a controlled release of the antibiotic, which could be noticed for at least 24 h of contact (Figure 4). This time period is very important for establishing a microbial infection since most device-associated infections occur at short time after the implantation procedure [56]. 

### 3.2. Physicochemical Characterisation of PLGA/Fe_3_O_4_@CEF Coatings

**Figure 5:**
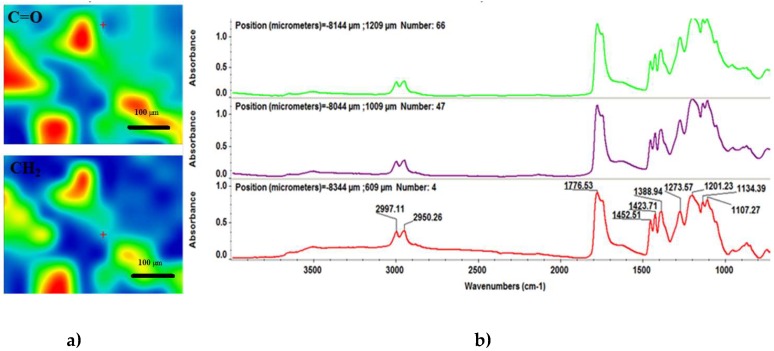
Infrared micrographs (**a**) and corresponding infrared spectra (**b**) of PLGA/Fe_3_O_4_@CEF dropcast coating.

**Figure 6:**
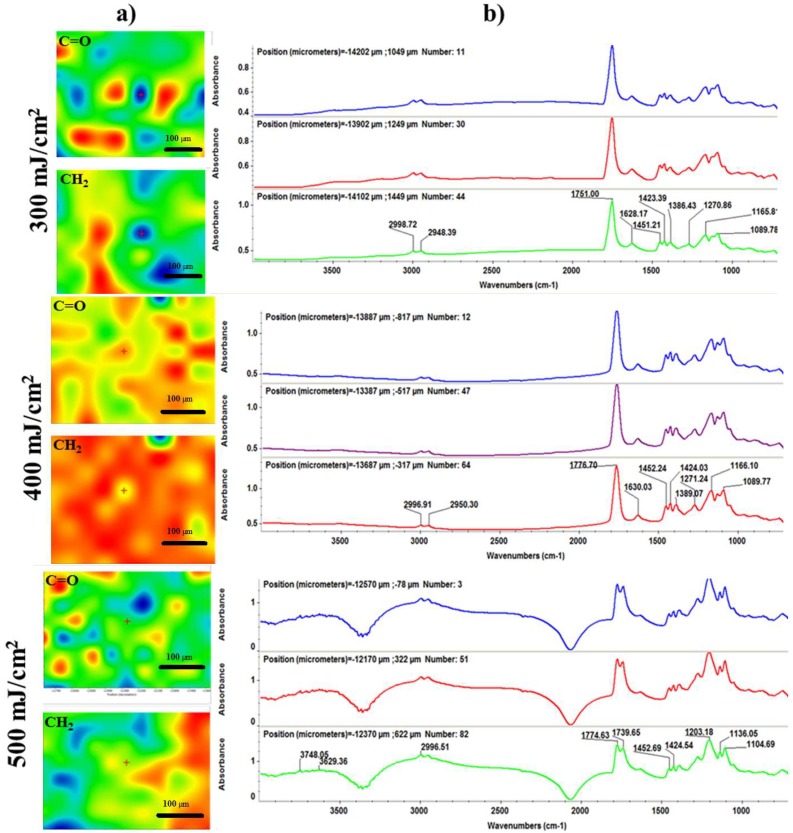
Infrared micrographs (**a**) and corresponding infrared spectra (**b**) of PLGA/Fe_3_O_4_@CEF coatings obtained at 300, 400, and 500 mJ/cm^2^ laser fluence.

To investigate the chemical composition and structural integrity of the synthesized coatings, we performed comparative infrared microscopy studies on both dropcast (Figure 5) and MAPLE (Figure 6) samples. In this respect, to attain the infrared maps we considered the stretching and asymmetric stretching vibrations of carbonyl and methylene functional groups, respectively, while the infrared spectra were collected by considering distinctive points on the concerned samples. As a general remark regarding the infrared microscopy results, the absorbance intensity of the collected infrared spectra is directly related to the colour changes within the resulted infrared maps, ranging from blue to red (corresponding to the lowest and highest intensity, respectively).

With respect to the infrared maps from Figure 6a, we can clearly notice the abundance of blue to green areas, which is a clear indication that the dropcast method resulted in non-uniform distribution of the PLGA/Fe_3_O_4_@CEF mixture onto the substrate. However, given the non-harmful transfer process of the composite mixture, we used the dropcast results for detailed infrared analysis. Regarding the absorbance spectra of the dropcast sample (Figure 6b), the broad band identified around 3500 cm^−1^ wavenumber is the result of overlapped free hydroxyl stretching from both organic compounds. The following double-humped peak (with maxima values at ~2997 and ~2950 cm^−1^) is the result of the asymmetric stretching vibrations of –CH_3_ and –CH– groups within the copolymer [57]. The intense vibrational peak identified at ~1776 cm^−1^ wavenumber is assigned to the overlapped symmetric stretching vibrations of C=O function, which is specific for both copolymer and lactam ring within the antibiotic [58,59]. The bending vibrations of ending amine groups within Cefepime may cause the very weak infrared signal positioned at ~1600 cm^−1^ wavenumber. The peak identified at ~1380 cm^−1^ may be assigned to the superposition of –OH stretching and –CH_3_ bending vibrations within the copolymer, while the infrared maxima positioned at ~1130 cm^−1^ may result from the overlapping of C–O and C–O–C asymmetric stretching vibrations within PLGA [60,61].

As one can notice from the IR maps depicted in Figure 5a (which were built by considering the ~2950 and ~1776 cm^−1^ wavenumbers corresponding to methylene and carbonyl chemical functions), a significant improvement—in terms of functional groups distribution—is reported for all the synthesized MAPLE samples, regardless the laser fluence value. Given the acknowledged advantages of the MAPLE technique regarding the stoichiometric transfer of various organic compounds [62,63,64] and the obtained data, one can thus support the idea that this particular laser processing strategy represented the proper choice for the synthesis of the composite materials within our study, with a particular remark on the middle laser fluence. Furthermore, if we also consider the corresponding infrared spectra of the laser processed samples (Figure 6b), the lowest laser fluence (300 mJ/cm^2^) resulted in no peak alteration or position modification for any of the previously identified absorbance maxima, but resulted in the least intense peaks, which may be related to the reduced amount of material transferred onto the substrate. When compared to the dropcast sample, the composite material processed by using the highest fluence (500 mJ/cm^2^) resulted in significant modification of the infrared maxima, due to the damage of the copolymer after laser beam interaction.

With respect to the MAPLE samples, the best results were undoubtedly obtained in the particular case of the middle laser fluence (400 mJ/cm^2^), which resulted in concurrent uniform distribution of the chemical functions onto the entire surface of the substrate and stoichiometric transfer of the PLGA/Fe_3_O_4_@CEF mixture. Regarding the presence of Fe_3_O_4_ within our materials, the infrared results evidence no additional bands when compared to the infrared data available for pristine Cefepime, suggesting thus only weak interactions established by hydrogen bonds between the amine terminal groups of the antibiotic and the abundant hydroxyl groups from the surface of magnetite. Moreover, when compared to the literature data regarding the infrared behaviour of pristine Cefepime and PLGA, no additional absorbance maxima are identified within our spectra—neither for the dropcast, nor for the MAPLE samples—suggesting thus the sole physical interactions established between the two compounds. By gathering the collected infrared data, one may conclude that the MAPLE technique enabled the successful synthesis of composite materials consisting in Cefepime-functionalized magnetite dispersed into PLGA-based matrix for the particular laser fluence of 400 mJ/cm^2^.

From the compositional point of view, the middle laser fluence (400 mJ/cm^2^) proved to be beneficial for the MAPLE synthesis of the PLGA/Fe_3_O_4_@CEF composite coatings. Therefore, for further investigations (both microstructural and biological), we selected only the samples obtained at this particular laser fluence.

**Figure 7:**
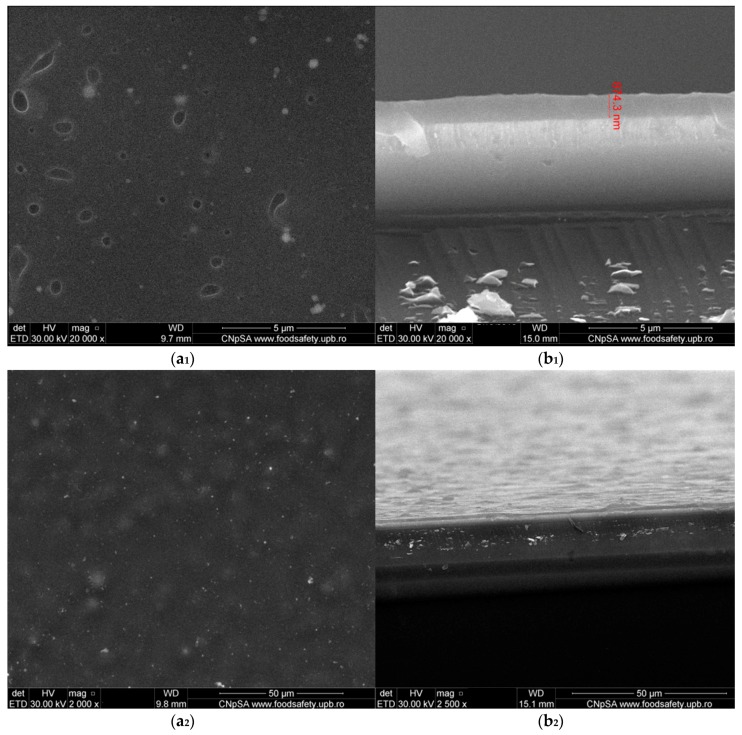
Plain view of the obtained MAPLE surface at different magnifications, (**a_1_,a_2_**), and cross section of the thin film revealing average thickness and (**b_1_,b_2_**) obtained through SEM analysis (at 20,000 magnification ) of PLGA/Fe_3_O_4_@CEF coating obtained at 400 mJ/cm^2^ laser fluence.

Relevant microstructural data about the as-synthesized PLGA/Fe_3_O_4_@CEF samples were collected from the SEM data, pictured in Figure 7. The plain view micrograph (Figure 7a) evidences the uniform distribution of the composite material, with no clear signs of physical damage due to laser interaction. Also, small cavities can be locally identified onto the entire surface, which might have resulted after the rearrangement of the material onto the substrate. Such particular “holey” aspect of the PLGA/Fe_3_O_4_@CEF coatings may represent the concurrent consequence of both colloidal suspensions used for MAPLE target preparation and wettability differences between the polymer matrix and the silicon substrate. As one can notice, the addition of functionalized magnetite nanoparticles encouraged the formation and uniform distribution of spherical nanosized aggregates within the entire PLGA matrix (the bright areas).

The cross section micrograph corresponding to our composite coatings (Figure 7b) confirms the formation of uniform and compact PLGA/Fe_3_O_4_@CEF coatings. Given the acknowledged advantages of MAPLE processing for organic and composite materials [65,66], the thickness of our composite coatings has reduced dimensional variation in the investigated areas and was evaluated below 1 μm.

### 3.3. Biological Evaluation of the PLGA/ Fe_3_O_4_@CEF Coatings

**Figure 8:**
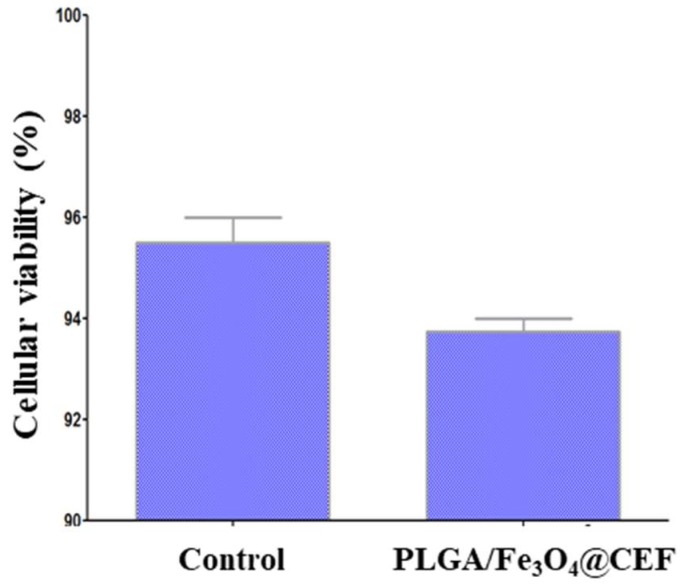
Viability of amniotic fluid-derived stem cells (AFSCs) after 72 h of treatment in the presence of PLGA/Fe_3_O_4_@CEF coating.

The obtained cellular viability results with respect to the quantitative biocompatibility of the PLGA/Fe_3_O_4_@CEF coatings are pictured in Figure 8.

In terms of quantitative biological evaluation of their biocompatibility, the results corresponding to our materials are comparable with the control specimens even after three days of treatment, since the cellular viability variation is not bigger than 2%. The increased biocompatibility corresponding to the PLGA/Fe_3_O_4_@CEF coatings (above 93%) suggest efficient adhesion and proliferation of the AFSCs in the presence of the proposed composite materials.

**Figure 9:**
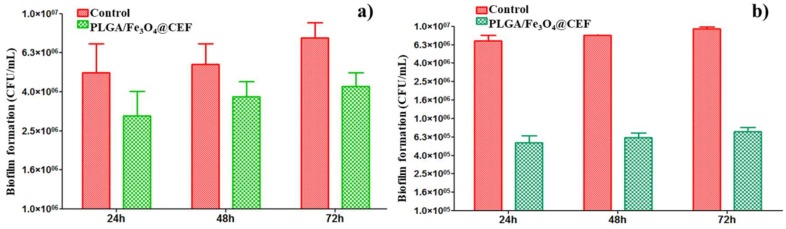
Biofilm development inhibition of *E. coli* (**a**) and *S. aureus* (**b**) in the presence of PLGA/Fe_3_O_4_@CEF coatings.

Given the bactericidal activity of Cefepime against clinical-interesting opportunistic bacteria, we experimentally assessed the efficiency of the PLGA/Fe_3_O_4_@CEF materials against biofilm development of Gram-negative (*Escherichia coli*) and Gram-positive (*Staphylococcus aureus*) strains, the obtained results being pictured in Figure 9.

As a general remark, one can notice that the PLGA/Fe_3_O_4_@CEF coatings resulted in a reduced development of bacterial biofilm, regardless the pathogen strain and the investigated period of time. In the case of *E. coli* (Figure 9a), we can observe a moderate and sustained efficiency of the composite coatings against the development of bacterial biofilm. This observation is applicable for all the investigated time intervals, since the obtained CFU/mL values decrease significantly. Such behaviour was quite expected, if we consider the acknowledged efficiency of Cefepime against the *Enterobacteriaceae* family. On the other hand, the PLGA/Fe_3_O_4_@CEF coatings proved a significant efficiency also against the formation of *S. aureus* biofilm (Figure 9b). Interesting fact, only a slight variation in terms of CFU/mL values can be noticed for both control and composite-coated substrates, regardless the evaluated incubation period. With a bacterial colonisation reduced with at least one order of magnitude, the PLGA/Fe_3_O_4_@CEF coatings proved to represent an efficient and sustained strategy against the Gram-positive biofilm formation. The fact that the antibiofilm effect can be observed for at least 72 h suggests that the antibiotic is released in a controlled manner by the obtained nanosystem and this activity potentate its antimicrobial effect, while maintaining it active for at least 3 days.

## 4. Discussion

We intended to obtain biocompatible materials with antibacterial effects to coat the available biomedical materials and devices that are highly susceptible to pathogenic contamination and colonization processes. Besides its utilization as the polymer matrix within the proposed coatings, PLGA was selected given its intrinsic biocompatibility and biodegradability, but also its hydrophilic behaviour and specific glass transition [67]. Under local temperature increase conditions (caused by the inflammatory processes that accompany physiological immune reactions), the PLGA matrix will enable the local release of antibiotic-coated nanoparticles, providing thus an increased antibacterial activity [68]. Moreover, magnetite nanoparticles were selected in this study thanks to the following motivation: (i) they can potentiate the therapeutic effects of sole antibiotic thanks to the specific nanosize-related properties, providing enhanced antibacterial activity at low drug concentrations; (ii) they can act as passive carriers of the antibiotic, since their sole release from the polymer matrix can result in an enhanced antibacterial efficiency; and (iii) they can act as active carriers of the antibiotic, since their eventual exposure to external magnetic fields can result in complete antibiotic release and, consequently, in enhanced antibacterial activity [69].

The successful encapsulation of broad-spectrum antibiotics within spherical chitosan-coated magnetite nanoparticles (CS-Fe_3_O_4_) with mean diameter of 50 nm was reported by Kariminia and co-workers. The obtained systems were evaluated as efficient nanocarriers for antibiotic loading (the drug loading efficiency was assessed above 99%), but also as pH-dependent and temperature-dependent nanostructures. Under body simulated conditions (pH = 7.4, 37 °C), a prolonged release (up to 5 days) of ~95% of the loaded antibiotic was reported. Moreover, the CS-Fe_3_O_4_ nanoparticles were evaluated as effective drug delivery systems under short-term low frequency ultrasound irradiation [70]. In a similar study, Dhivya and the co-workers (2016) synthesized chitosan-coated iron oxide nanoparticles with 20 nm mean particle size and weak ferromagnetic behaviour at room temperature conditions. By using two distinctive quantification protocols, the CS-Fe_3_O_4_ nanoparticles were assessed as efficient dose-dependent inhibitors against *Escherichia coli* bacterial development after 24 h of treatment [71].

In the particular case of natural-derived bioactive substances, several studies report the beneficial conjunction between the inorganic biocompatible Fe_3_O_4_ and essential oils (EOs), the magnetite nanoparticles acting both as a stabilization agent for the volatile compounds and as a functional enhancing carrier. Grumezescu and co-workers reported the successful use of a nanofluid based on Fe_3_O_4_ core, oleic acid shell and usnic acid extra-shell (FeOAU) for the potentiated activity of usnic acid against the development of *Staphylococcus aureus* biofilm for up to 72 h of in vitro evaluation [72]. By using Fe_3_O_4_/oleic acid core/shell nanoparticles with 20 nm mean particle size and a modified immersion—solvent evaporation protocol, Chifiriuc and co-workers developed thin pellicles of Fe_3_O_4_/oleic acid/*Rosmarinus officinalis* essential oil onto the surface of commercial catheter sections. The as-modified medical devices proved to significantly affect the biofilm development of *Candida albicans* and *Candida tropicalis* fungal strains, being efficient both during initial contamination and subsequent colonisation stages [73]. A similar study reported the synthesis of 10 nm sized magnetite nanoparticles functionalized with sodium stearate (MNP@18) for the superficial entrapment of *Satureja hortensis* essential oil (MNP@18-SH). Commercial wound dressings modified with the as-obtained MNP@18-SH nanosystems significantly impaired cellular adhesion (24 h) and mature colonies (48 and 72 h) of *Candida albicans*, resulting thus in an efficient strategy against fungal biofilm formation [74]. The immersion—solvent evaporation strategy was also used by Bilcu and co-workers to modify the surface of polyvinyl chloride catheter sections with pellicles of Fe_3_O_4_@C_14_-EOs (magnetite core, myristic acid shell and essential oil extra-shell). The pellicles containing vanilla essential oil significantly affected initial adherence of *Staphylococcus aureus* bacteria (24 h) and subsequent mature biofilm formation (48 h), but only affected the initial contamination stage of *Klebsiella pneumoniae* (24 h). In the case of both pathogens, the catheter sections modified with Fe_3_O_4_@C_14_ nanoparticles loaded with patchouli and ylang-ylang EOs impaired only the initial bacterial contamination stages [75].

By using a modified co-precipitation method, Grumezescu and co-workers [47] have successfully synthesized polyhedral 5 nm sized magnetite nanoparticles coated with a thin and uniform layer of commercial Kanamycin antibiotic (Fe_3_O_4_@KAN). After a 2 days in vivo treatment performed on albino mice, the as-obtained nanosystems exhibited non-preferential affinity for most vital organs, but specific histological alterations with respect to the pulmonary tissue, suggesting their potential use for non-conventional treatment of lungs conditions. In addition, thin coatings based on Fe_3_O_4_@KAN nanoparticles (obtained by MAPLE technique) were assessed as biocompatible materials in the presence of human endothelial cells cultures, significant quantitative and qualitative cellular compatibility results being reported for up to 3 days of treatment. Moreover, the Fe_3_O_4_@KAN coatings were assessed as an efficient strategy to reduce biofilm development, since the assays performed against *E. coli* and *S. aureus* strains revealed an important time-related impairment of bacterial contamination and colonisation after 72 h of treatment [47].

As a result of the alarming phenomenon of multidrug-resistant microorganisms and ineffective or nonspecific conventional antimicrobial therapy, a remarkable interest has been straightened towards the development of novel antimicrobials. In this respect, the functional re-evaluation of commercial or natural-derived bioactive substances by means of nanosystems (including inorganic, organic, composite and hybrid nanostructured materials) seems to represent an attractive and versatile option for novel patient-oriented antimicrobial therapy.

The obtained PLGA/Fe_3_O_4_@CEF coatings possess promising potential for short to mid-term biomedical applications, being efficient in inhibiting microbial colonization and biofilm formation for at least three days, covering the critical initial time scale which is often incriminated for devide-associated infections. The Fe_3_O_4_@CEF composite is a promising material for designing novel biocompatible coatings with sustained efficiency during the early and middle stages of bacterial colonisation, has a good biocompatibility and a low tendency to cluster in vital organs, aggregates they being visible only in the spleen.

## 5. Conclusions

The present study reports the successful synthesis of biocompatible PLGA/Fe_3_O_4_@CEF composites as promising antibacterial coatings for implantable devices. The selected chemical approach resulted in the successful synthesis of core/shell Fe_3_O_4_@CEF structures, consisting in crystalline and spherical shaped magnetite core coated with a uniform and reduced amount of antibiotic layer. The MAPLE technique enabled the effective transfer of nanostructured PLGA/Fe_3_O_4_@CEF material and resulted in the synthesis of stoichiometric and uniform composite thin coating. The performed in vitro and in vivo assays evidenced a prolonged biocompatibility of the PLGA/Fe_3_O_4_@CEF coatings, while the antibacterial tests emphasized a sustained and efficient inhibition of bacterial biofilm development. One can conclude that the proposed composite coatings based on PLGA and Cefepime-functionalized magnetite nanoparticles are suitable materials for the unconventional treatment of implantable devices surface, in terms of enhanced biocompatibility and reduced bacterial colonization.
